# The Well‐Being of Slum Dwellers Are Associated With WaSH‐Related Factors: A Cross‐Sectional Study From India

**DOI:** 10.1002/hsr2.70811

**Published:** 2025-07-09

**Authors:** P. Padma Sri Lekha, E. P. Abdul Azeez, B. Latha Lavanya, V. Kalyani, Manoj Mathew, S. Giri Prasath, J. Leo Joshwin, U. Nithyasree

**Affiliations:** ^1^ School of Social Sciences and Languages Vellore Institute of Technology Vellore Tamil Nadu India; ^2^ MEASI Institute of Management Chennai Tamil Nadu India; ^3^ Department of Social Work Rajagiri College of Social Sciences Cochin Kerala India; ^4^ Department of Social Work Kalinga University Raipur Chhattisgarh India

**Keywords:** India, KAP, mental Health, sanitation‐related quality of life, WaSH, water Insecurity experience, well‐being

## Abstract

**Background and Aims:**

The adverse health outcomes due to unsafe Water, Sanitation, and Hygiene (WaSH) are a public health concern in low‐and middle‐income countries. However, evidence on how WaSH is associated with psychological outcomes is minimal. Insights on this association would help address the well‐being of the slum population. Therefore, this study aimed to understand the association between knowledge, attitude and practice (KAP) of WaSH, water insecurity experiences, and sanitation‐related quality of life on the well‐being of individuals living in slums.

**Methods:**

We used the KAP of WaSH, water insecurity experience scale, sanitation‐related quality of life scale, and WHO well‐being index to collect data from adult slum dwellers aged 18 years and above from Vellore, Tamil Nadu, India. We adopted a cross‐sectional study design and employed a systematic sampling procedure to select the households. The data was collected through a face‐to‐face household survey (*n* = 258; Male = 171; Female = 87). We employed a multiple regression model to understand the association of various factors with well‐being.

**Results:**

The results indicated that a positive attitude toward WaSH and higher water insecurity experiences significantly reduced the well‐being of the slum dwellers. However, good WaSH practices (*β* = 0.37; 99% CI = 0.23–0.51) and sanitation‐related quality of life (*β* = 0.38; 99% CI = 0.25–0.50) led to good well‐being among slum‐dwellers. Also, the presence of toilets significantly contributed to the increase in well‐being and sanitation‐related quality of life. In summary, the accessibility to water sources, appropriate sanitation facilities, and hygiene practices increased the likelihood of well‐being among slum‐dwellers.

**Conclusion:**

The results suggest that promoting adherence to proper WaSH practices, enhancing water security, and providing availability and access to toilet facilities for individuals living in slums are important to improving their well‐being.

## 1 Introduction

Safe water, sanitation, and hygiene (WaSH) are essential for a healthy life. Although safe WaSH is a basic need, many individuals and communities lack it globally. As reported by the World Health Organization (WHO), 1.7 billion people use contaminated drinking water sources, and 2 billion people live in water‐stress countries as of 2021, with the prediction of an increase in water stress soon [1]. The unsafe water resources would pave the way for various detrimental physical, psychological, and social effects among individuals [2]. A similar condition was observed with sanitation, as about 1.5 billion people were deprived of basic sanitation facilities [3]. These factors influence the daily activities of individuals and health, making it a public health concern, especially in low‐ and middle‐income countries.

A global analysis with selected disease outcomes estimated that 1.4 million deaths and 74 million disability‐adjusted life years (DALYs) could have been prevented with safe WaSH and the disease outcomes as diarrhea, acute respiratory infection, undernutrition, and soil‐transmitted helminthiasis were attributed to unsafe WaSH [4]. In addition, households with unsafe WaSH practices had increased occurrence of diarrhea, outpatient visits, malaria, and anemia [5, 6, 7, 8, 9]. However, a neighborhood with improved water and good sanitation coverage reduced these, including malnutrition [10]. This points to the importance of community‐level changes in improving safe water usage and sanitation. The water and sanitation factors impacted children's cognitive development [11, 12] and school attendance [13]. Along with this, the poor sanitation facilities negatively affected psychological and social well‐being, as individuals experienced or perceived a lack of safety and privacy during open defecation [14]. The compromised sanitation facilities led to physical and sexual assaults with bodily injuries, especially among girls and women [15, 16], further tampering with their mental health and well‐being.

The geographical, economic, and sociocultural disparities made individuals in lower‐ and middle‐income countries, especially those residing in rural areas or urban slums, highly vulnerable to health and psychological issues [3]. Poor WaSH is attributed to a lack of Knowledge, Attitude, and Practice (KAP) of WaSH among individuals [17, 18] due to their lower education and poverty. In addition, the sociocultural aspects played a crucial role in adapting to safe water and sanitation facilities, as studies pointed out how the outdated traditional sociocultural beliefs and practices restrained individuals from using good WaSH facilities [16, 19]. A study in north India identified the prevalence of open defecation even though they had a functional latrine, attributing it to attitudes [20, 21]. Switching to latrine use from open defecation was significantly low and was primarily determined by social context [22]. Furthermore, a study suggested the higher prevalence of waterborne diseases among older adults in India, attributing the condition to their lack of basic hygiene [23]. These factors are prominent in low and middle‐income countries like India. A study in a semi‐urban setting identified that 43.5% had poor water handling practices, unimproved sanitation, and an increased incidence of diarrhea [24]. Also, a study in rural Odisha identified poor WaSH practices and their association with acute and chronic health conditions [25]. Further, the hygiene practices and sanitation were determined by socioeconomic status and place of residence [26].

KAP of WaSH prevalence, water insecurity experience, and sanitation have been strongly associated with negative health outcomes. However, a significant gap exists in the literature addressing well‐being in this context [27]. Further, understanding these aspects will be insightful in the context of India since the country is home to a large proportion of slum‐dwellers. Therefore, this study aimed to understand the role of KAP of WaSH, water insecurity experiences, and sanitation‐related quality of life on the well‐being of individuals living in slums in Vellore, India.

## 2 Methods

### 2.1 Study Locality and Data

This study was conducted in Vellore City, Tamil Nadu, India. Vellore City has 49 registered slums, constituting 10,750 households. Most slum localities have poor water and sanitation facilities, making it an ideal setting for the present study. We adopted a cross‐sectional study design, and the data were collected from three slum localities of Vellore City through systematic sampling. We conducted a face‐to‐face survey with one of the residents at every fifth household in the three slum areas. The data was collected between the period of September to December 2023.

The sample size was calculated using the formula:

$$n=Z_{\alpha /2}^{2}\;p\left(1-p\right)/\varepsilon^{2},$$

where *Z*_*α*/2_ = 1.96; *p* = the proportion of poor sanitation practice (81%) [28]; *ε* = margin of error (0.05). By substituting the values, the minimum sample size required for significant power was 237. The survey was completed by a household member, *n* = 258 (Male = 171; Female = 87), aged 18 years and above.

### 2.2 Measures/Tools

#### 2.2.1 Outcome Variable

##### 2.2.1.1 Well‐Being

The well‐being of adults living in the slum was assessed using five items WHO Well‐Being Index [29] with a Likert scale response of 5 (*all the time*) to 0 (*at no time*). The score ranges from 0 to 25, and a high score indicates better well‐being. The participants were asked to respond to these questions in the context of WaSH.

#### 2.2.2 Predictor Variables

##### 2.2.2.1 Knowledge, Attitude, and Practice (KAP) on WASH

We used a scale employed in Berhe et al. [17] study with 11 items under knowledge and 20 and 13 items under attitude and practice of WASH, respectively. The scoring pattern differed for all three domains, scoring 1 for correct answers and 0 for wrong answers under knowledge and practice. At the same time, the attitude had a 5‐point Likert response with 1 (*Strongly Disagree*) to 5 (*Strongly Agree*). A higher score in KAP indicates good knowledge, a favorable attitude, and good practice on WASH.

##### 2.2.2.2 Water Insecurity Experience

The Household Water Insecurity Experience Scale (HWISE) developed by Young et al. [30] for low and middle‐income countries with twelve items were used to assess water insecurity experience among slum‐dwellers. The scale had a response pattern of 0 (*Never*) to 3 (*Always*), and the score ranged from 0 to 36. A high score indicates increased water insecurity experience in the past 4 weeks.

##### 2.2.2.3 Sanitation‐Related Quality of Life

This was assessed using the five items scale developed by Ross et al. [31] with a response pattern of 0 (*Never*) to 3 (*Always*). The score ranges from 0 to 15, with a high score indicating good sanitation‐related quality of life.

##### 2.2.2.4 Socio‐Demographic Variables

These included age, gender, education, and the presence of a toilet and a separate kitchen.

##### 2.2.2.5 Statistical Analysis

We used JAMOVI 2.3.21 to conduct all the analyses. First, a descriptive and correlational analysis was run to understand the characteristics and associations between the variables. Second, we used a multiple regression model to understand the role of KAP on WaSH, water insecurity experience, and sanitation‐related quality of life on the well‐being of slum dwellers. Third, a linear regression was conducted to observe the association of the availability of toilets on well‐being and sanitation‐related quality of life. All the statistical analyses were two‐tailed and reported at 95% and 99% confidence levels.

## 3 Results

The descriptive characteristics of categorical variables are presented in Table 1. The sample had a higher proportion of males (66.3%), and above 50% of the participants had primary or secondary education. In addition, 59.7% had a family size of 4 and above, and for 40.7% of households, the monthly income ranged between Rs. 10,001 and 20,000. As expected, 80.6% had no toilet, and 54.3% had no separate kitchen.

**Table 1:** Descriptive characteristics of categorical variables of the sample (*N* = 258).

Variable	Frequency	%
Gender		
Male	171	66.3
Female	87	33.7
Education		
Primary	68	26.4
Secondary	81	31.4
Higher secondary	69	26.7
Graduation and above	40	15.5
Family size		
Single‐member	5	1.9
2 Members	18	7.0
3 Members	81	31.4
4 and above	154	59.7
Monthly family income		
Rs.5000–10,000	96	37.2
Rs.10,001–20,000	105	40.7
Rs.20,001 and above	57	22.1
Presence of toilet		
No	208	80.6
Yes	50	19.4
Separate kitchen		
No	140	54.3
Yes	118	45.7

Table 2 presents the descriptive and correlation analysis of continuous variables. The mean age of the study sample was 42 years (Standard deviation [SD] = 12.6) with an average score of 9.23, 73.7, and 5.09 on Knowledge, Attitude, and Practice of WASH, respectively. From the mean cutoffs, it was evident that 17% had poor knowledge, 45% and 53% had unfavorable attitudes and poor practice of WASH, respectively. This suggests a gap between knowledge and practice of WASH among slum dwellers. In addition, the mean on the water insecurity experience scale, sanitation‐related quality of life and well‐being was 9.52, 4.81, and 12.2, respectively. Considering the mean cutoff, it is evident that 58% of slum dwellers had higher water insecurity experience. In addition, more than 50% of individuals had poor sanitation‐related quality of life and well‐being. Further, Knowledge (*r* = 0.18; *p* < 0.001) and Practice (*r* = 0.64; *p* < 0.001) on WASH and sanitation‐related quality of life (*r* = 0.67; *p* < 0.001) were positively associated with well‐being while water insecurity experiences (*r* = −0.33; *p* < 0.01) had a negative relationship with it.

**Table 2:** Descriptive and correlational analysis of continuous variables.

Variables	Mean	SD	1	2	3	4	5	6
Age	42.0	12.6						
1. Knowledge	9.23	1.51	1					
2. Attitude	73.7	8.12	0.54**	1				
3. Practice	5.09	3.08	0.41**	0.33**	1			
4. WIE	9.52	5.67	−0.19*	−0.21**	−0.44**	1		
5. SAN‐ QOL	4.81	4.72	0.23**	0.06	0.71**	−0.29*	1	
6. Well‐being	12.2	4.02	0.18**	−0.01	0.64**	−0.33**	0.67**	1

Abbreviations: SAN‐QOL, sanitation‐related quality of life; SD, standard deviation; WIE, water insecurity experience.

*p < 0.01; **p < 0.001.

A multiple regression analysis with well‐being as an outcome was run to understand the role of these predictors (Table 3). It is important to note here that the knowledge and practice of WaSH showed a positive correlation with well‐being, while attitude was not significantly associated (as observed in Table 2). However, accounting for knowledge, practice, water insecurity experience, and sanitation‐related quality of life in multiple regression model, a positive attitude towards WASH reduced the likelihood of well‐being (*β* =−0.09; 99% CI = −0.30 to −0.09) while a good practice increased it (*β* = 0.37; 99% CI = 0.23–0.51). This could be possibly due to the gap between cognitive aspect (knowledge and attitude) and behavior (practice). In addition, the water insecurity experience (*β* = −0.11; 95% CI = −0.21 to −0.02) reduced the likelihood of well‐being. However, good sanitation‐related quality of life increased the probability of well‐being (*β* = 0.38; 99% CI = 0.25–0.50), suggesting the importance of appropriate sanitation facilities. In summary, sufficient access to water, appropriate sanitation, and good hygiene practices promoted the likelihood of well‐being. At the same time, the gap between attitude and WaSH‐related practices reduced the probability of well‐being among slum dwellers. Interestingly, more than 50% of the variance in well‐being among slum dwellers was associated with by these factors related to WaSH.

**Table 3:** Result of multiple regression with well‐being as an outcome.

Variables	*β*	95% confidence interval		*p*
		Lower limit	Upper limit	
Knowledge	0.02	−0.08	0.12	> 0.05
Attitude	−0.09	−0.30	−0.09	< 0.001
Practice	0.37	0.23	0.51	< 0.001
WIE	−0.11	−0.21	−0.02	< 0.05
SAN‐QOL	0.38	0.25	0.50	< 0.001

*Note:* Summary: *R* ^2^ = 0.54; *F*(5, 252) = 60.2, *p* < 0.001.

Abbreviations: SAN‐QOL, sanitation‐related quality of life model; WIE, water insecurity experience.

**Figure 1:**
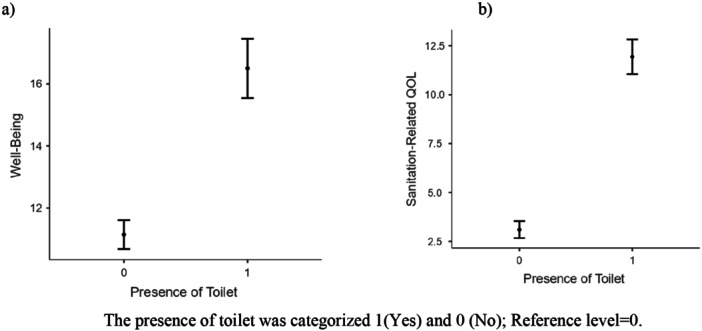
The estimated marginal mean plot for presence of toilet with well‐being and sanitation‐related quality of life. The presence of toilet was categorized 1(Yes) and 0 (No); Reference level = 0. *Note(a)*: *β* = 1.33, 99% CI = 1.07 to 1.60; Model Summary: *R* ^2^ = 0.28; *F*(1, 256) = 98.5, *p* < 0.001. *Note(b)*: *β* = 1.87, 99% CI = 1.67 to 2.08; Model Summary: *R* ^2^ = 0.55; *F*(1, 256) = 314, *p* < 0.001.

A linear regression was conducted to understand the likelihood of well‐being and sanitation‐related quality of life among individuals with and without a toilet (Figure 1). The presence of a toilet promoted better well‐being and sanitation‐related quality of life compared to individuals who do not have it (*β* = 1.33, 99% CI = 1.07 to 1.60 and *β* = 1.87, 99% CI = 1.67 to 2.08, respectively). In addition, 55% of the variance in sanitation‐related quality of life was related to the availability of toilets among slum dwellers.

## 4 Discussion

The present study tried to understand the role of KAP on WaSH, water insecurity experiences, and sanitation‐related quality of life on well‐being among slum dwellers. The results of this study suggested a positive association between knowledge, practice, and well‐being. Minuscule studies have addressed this association. However, the literature suggests an association between poor knowledge and practice of WaSH and vulnerability to infectious diseases [4, 17, 32], which could decrease the well‐being of the individuals. Similarly, in line with the current study results, a study conducted in Bangladesh reported that 90% had knowledge about hand washing, but the practice was poor [33]. Further, a study among mothers of children under 5 years in rural India pointed to the poor knowledge of water purification, but the knowledge of washing hands after defecation was good [34]. Good knowledge about WaSH would enhance well‐being by reducing uncertainty in their practice. Also, the practice is not only determined by knowledge, as various psychosocial and socioeconomic factors influence the WaSH practices [35, 36].

Interestingly, the multiple regression model results indicate that a favorable attitude toward WaSH reduced the likelihood of well‐being while good practice enhanced it. The role of attitude and practice on well‐being has not been the focus of the WaSH literature, making this study one of its kind and significantly addressing the gap in this context. Moreover, the present results can be explained by the cognitive dissonance theory by Festinger [37], which states that individuals experience psychological discomfort when they identify an inconsistency in their beliefs or between their beliefs and behaviors. In the present study, an inconsistency can be observed regarding favorable attitudes toward WaSH and their practice, which could significantly reduce their well‐being. Although individuals had the right attitude towards WaSH, they could not implement it due to the lack of appropriate facilities to follow good sanitation and hygiene practices. This inconsistency can pave the way for dissonance, decreasing their well‐being. A study exploring the nature and characteristics of cognitive dissonance identified lower pleasure among individuals in dissonance conditions over their counterparts [38]. Nevertheless, a good practice consistent with the attitude could lead to better well‐being. However, the literature does not directly link WaSH practice with well‐being. Earlier studies point to the essential negative impact of poor hygiene and sanitation practices on health [39, 40], which could contribute to poor well‐being.

A higher water insecurity experiences reduced the likelihood of well‐being significantly, but a good sanitation‐related quality of life promoted it among slum‐dwellers in this study. On the same line, the previous studies pointed to the essential role of water in mental health, as water expenditures were positively associated with food insecurity and perceived stress [41]; water insecurity was also associated with emotional distress and well‐being [27]. In addition, studies have identified a significant association between water insecurity and psychological stress [42], along with the risk of injury during water fetching from unimproved sources, and chronic stress [43]. These factors related to water insecurity can decrease well‐being among individuals. Similarly, earlier studies identified the importance of appropriate sanitation facilities in well‐being as individuals were concerned about privacy and safety [14, 44].

The present study identified toilet availability as an essential contributor to well‐being among slum dwellers, as it enhanced well‐being and sanitation‐related quality of life compared to their counterparts without toilets at home. A study has identified that sanitation facilities are crucial to the quality of life and mental well‐being [44]. In addition, earlier studies have established that the availability of toilets is important, especially to girls' and women's well‐being [45] and overall quality of life [16], and to reduce violence against them [46]. However, the literature indicated minimum usage of toilets even in their availability [47], and the shift to appropriate sanitation facilities was poor [22], indicating a problematic attitude. This study uniquely contributes to understanding how WaSH‐related factors, especially the availability of toilets and sanitation‐related quality of life, influence the well‐being of people living in resource‐poor settings in India. Although this study is unique in its contribution, it has certain limitations. First, the study does not explain the causal relationship between the variables, as it is cross‐sectional. Second, this study addressed only well‐being as a psychological factor and did not consider other aspects.

## 5 Conclusion

The present study assessed the association of KAP of WaSH, water insecurity experiences, and sanitation‐related quality of life and well‐being among slum dwellers. The results show that the favorable attitude of WaSH and higher water insecurity experiences reduced the likelihood of well‐being. However, good practice and sanitation‐related quality of life increased the possibility of well‐being among slum‐dwellers. In addition, the presence of toilets significantly contributed to good well‐being and sanitation‐related quality of life. Interestingly, there exists a gap between WaSH knowledge and practice. Future intervention programs should focus on enhancing the appropriate practice to reduce disease incidence and promote well‐being. Further, it is essential to address the water and sanitation‐related insecurity experiences as they lead to day‐to‐day stress, increase the incidence of disease, and negatively impact well‐being. Initiatives should be taken to promote water security as it is a prime requirement for sanitation and hygiene. There is also a need for toilets in more than 80% of the households in the study localities. The policies should focus not only on toilet construction but also on its utilization. The future programs must promote toilet utilization among individuals living in slums to improve their well‐being. Future research can focus more comprehensively on the psychological and mental health outcomes of WaSH and its related aspects.

## Author Contributions

**P. Padma Sri Lekha:** conceptualization, methodology, data curation, investigation, project administration, writing – original draft, formal analysis. **E. P. Abdul Azeez:** conceptualization, methodology, data curation, supervision, investigation, writing – review and editing, project administration, resources. **B. Latha Lavanya:** conceptualization, data curation, project administration. **V. Kalyani:** conceptualization, formal analysis, supervision. **Manoj Mathew:** investigation, validation, supervision, project administration, data curation. **S. Giri Prasath:** data curation. **J. Leo Joshwin:** data curation. **U. Nithyasree:** data curation.

## Ethics Statement

All procedures performed in this study involving human participants followed Helsinki's declaration. The Research Ethics Committee of the Kalinga University, Raipur, India, granted the ethical approval for this study. Written informed consent was obtained from all the study participants.

## Conflicts of Interest

The authors declare no conflicts of interest.

## Transparency Statement

The lead author E. P. Abdul Azeez affirms that this manuscript is an honest, accurate, and transparent account of the study being reported; that no important aspects of the study have been omitted; and that any discrepancies from the study as planned (and, if relevant, registered) have been explained.

## Data Availability

The data that support the findings of this study are available on request from the corresponding author. The data are not publicly available due to privacy or ethical restrictions.
